# QTL Mapping for Seed Germination Response to Drought Stress in *Brassica napus*

**DOI:** 10.3389/fpls.2020.629970

**Published:** 2021-02-09

**Authors:** Mahmoud Gad, Hongbo Chao, Huaixin Li, Weiguo Zhao, Guangyuan Lu, Maoteng Li

**Affiliations:** ^1^Department of Biotechnology, Collage of Life Science and Technology, Huazhong University of Science and Technology, Wuhan, China; ^2^School of Agricultural Sciences, Zhengzhou University, Zhengzhou, China; ^3^Faculty of Biology and Food Engineering, Guangdong University of Petrochemical Technology, Maoming, China

**Keywords:** *Brassica napus*, seed germination, drought tolerance, QTL analysis, candidate genes

## Abstract

Drought stress is one of the most environmental abiotic stresses affecting seed germination and crop growth. In the present study, the genetic characteristics of seed germination under drought stress in a *Brassica napus* double haploid population were analyzed. Five germination-related indexes, including germination percentage (GP), root length (RL), shoot length (SL), fresh weight (FW), and root-to-shoot length ratio (R/S) under control and drought stress, were calculated, and the drought stress index (DSI), including DSI-GP, DSI-RL, DSI-SL, DSI-FW, and DSI-R/S, was determined using the quantitative trait loci (QTLs) analysis based on high-density genetic linkage map. The phenotypic analysis indicated that the R/S is an effective morphological trait in the determination of drought tolerance in the seedling stage. Thirty-nine identified QTLs were observed for these traits and then integrated into 36 consensus QTLs, in which 18 QTLs were found to affect the DSI of four traits (GP, RL, SL, and R/S). Based on the co-linearity between genetic and physical maps of *B. napus*, 256 candidate genes were detected, and 128 genes have single-nucleotidepolymorphisms/insertion–deletion (SNP/InDel) variations between two parents, some of which were associated with the drought stress tolerance (for example, *BnaC03g32780D*, *BnaC03g37030D*, and *BnaC09g27300D*). The present results laid insights into drought tolerance and its genetic bases in *B. napus*.

## Introduction

Worldwide, plants live in permanently changing environments that are often undesirable or stressful for growth and development. These unfavorable conditions include abiotic stresses, such as drought, heat, nutrient deficiency, cold, and salinity, and biotic stresses, such as herbivore attack and pathogen infections. One of the most important abiotic stresses impairing seed germination and yield in global agriculture is drought, which affects 40% of the world’s land area (Zhang et al., 2014).^1^ Drought significantly affects seed germination, leading to decrease of plant density and yield. In the future, it is expected that drought will probably increase in certain areas of the world. Unfortunately, global climate change could lead to increased drought in many parts of the world and thus could have a major impact on crops (Trenberth et al., 2013). Therefore, there is an urgent need to produce crop varieties capable of adapting to such conditions and maintaining high levels of productivity. These challenges have prompted scientists to do more to improve the ability of crops to resist such harsh conditions (Zhang et al., 2014).

Rapeseed (*Brassica napus* L., AACC, 2*n* = 4*x* = 38), which belongs to *Brassicaceae*, is one of the important crops, with soybean and oil palm considered as the largest oil crops in the world (Zhang D. et al., 2015). It is very sensitive to drought stress, which leads to severe yield reduction (Wan et al., 2009). The development of varieties with high tolerance to drought and drought-related stress could maintain the high yield production in some cases (Wan et al., 2009).

The genetic dissection of the quantitative traits that control the adaptation of crops to adverse conditions is a prerequisite for the development of many effective approaches, which aimed to improve the ability of accommodating itself to adversity and enhance yield production under abiotic stress (Li et al., 2014). It is worth mentioning that the performance of crops represents the final result of interactions between thousands of genes and environmental conditions as well as culture practices (Collins et al., 2008), and it is clear that analyzing quantitative trait loci (QTLs) is an effective method for dissecting the complex quantitative traits, which has been widely used in many crops, such as in wheat (Wang et al., 2018), rapeseed (Zhou et al., 2014), common bean (Sandhu et al., 2018), and peanut (Zhao et al., 2016). As QTLs were correlated with phenotype variation, the corresponding loci could be amplified and therefore used for phenotypic improvement (Raboanatahiry et al., 2017). QTL map studies for drought tolerance were conducted in many crops including *Arabidopsis*, rice, wheat, and soybean. However, the use of low marker density genetic linkage map was not able to reveal the comprehensive genetic diversity of drought tolerance in rapeseed (Li et al., 2014). Despite the progress made in the plant’s response to drought stress, many genetic bases of drought tolerance in rapeseed has yet to be clarified (Li et al., 2014; Zhang, 2015; Zhao et al., 2016; Liang et al., 2019). There has been significant progress in understanding the signaling process that controls plant resistance to drought from perception of signals to cellular mechanisms (Seki et al., 2007), and it was revealed that the signal pathways including many common factors, such as abscisic acid, photosynthesis, and reducing ROS contents under drought, were involved in drought resistance (Jackson et al., 2003; Li et al., 2006; Dudziak et al., 2019; Liang et al., 2019).

Recently, Chao et al. (2017) have developed a high-density genetic linkage map with 3207 markers based on the KN double haploid (DH) population. In the present study, the germination-related traits including germination percentage (GP), root length (RL), shoot length (SL), fresh weight (FW), and root-to-shoot length ratio (R/S), as well as the drought stress index (DSI) of these traits, were used to dissect the genetic basis of germination response under drought stress based on QTL mapping in *B. napus*, and candidate genes within the QTLs associated with drought tolerance during seed germination were identified.

## Materials and Methods

### Plant Material and Genetic Linkage Map

The segregation DH population, named KN, was used in this experiment. The KN DH population was derived from a cross between the two parental lines KenC-8 and N53-2 first constructed by Wang et al. (2013). The high-density single-nucleotide polymorphisms (SNP)-based linkage map, including 3106 SNP bins (including 17,978 SNPs) and 101 non-SNP markers (SSR and STS) with an average genetic distance of 0.96 cM between adjacent loci constructed by Chao et al. (2017), was used for QTL mapping in the current study.

### Experimental Design and Evaluation of the Phenotypic Indexes

The seeds of two parents (KenC-8 and N53-2) and KNDH lines were disinfected firstly with 70% ethanol and prepared for seed germination test. Germination test was carried out in a seed germination box (L × W × H = 12 cm × 12 cm × 5 cm). Firstly, two layers of filter paper were placed on the bottom of the dish and then 15 ml of ddH_2_O (control) or 15% BEG6000 solution (drought stress treatment) was added (Michel and Kaufmann, 1973). Fifty seeds were sowed in each dish and covered and then the dishes were placed in the growing room. The germination rate was calculated after 7 days of cultivation, and three representative single seedlings from each line were selected to measure biological indicators including GP, RL, SL, FW, and R/S, and three biological repeats were implemented for the survey. Drought resistance was measured by the drought resistance coefficient (ratio of treatment to control) and DSI = traits under drought/traits under control × 100% (Zhang J. et al., 2015).

### QTL Analysis

Quantitative trait locus analysis was performed as described by Wang et al. (2013). Windows QTL Cartographer 2.5 software was employed to identify the QTLs using the composite interval mapping (CIM) method (Zeng, 1994). The window size was 10 cM and the scan walking speed was 2 cM for the CIM method. The logarithm of odds (LOD) thresholds of QTL were determined by 1,000 permutation tests at the 95% confidence level (Doerge and Churchill, 1996). The BioMercator V4.2 program (Arcade et al., 2004) was used to integrate the QTL detected under control and drought stress with overlapping CIs into consensus QTL. The integration and nomenclature of QTLs were determined according to the method described by Wang et al. (2016). “K” was added at the end of the QTL name for QTLs detected under control to distinguish them from those detected under drought stress; for example, two QTLs, qRLK-6-1, and qSLK-11-1, were associated with root and SL under control, while two QTLs, qRL-9-1, and qSL-2-1, were associated with RL and SL under drought stress, respectively.

### Identification of Candidate Genes

Candidate genes were detected according to the description of Cai et al. (2014). The physical and genetic map alignment relationship was confirmed using all SNPs in the genetic map, and 50 per probe sequence were obtained by Illumina Inc., as a query in the detection of homolog loci by the NBCI-Blstn local program against the “Darmor-*bzh*” reference *B. napus* genome (Chao et al., 2017; Rotmistrovsky et al., 2004). The detected regions on the genome that align with the confidence interval of QTLs were considered as QTL regions, and the genes located within the QTL were defined as candidate genes of the QTL, as described by the method of Chao et al. (2017). SNP/insertion–deletion (InDel) variations of candidate genes were analyzed according to the re-sequencing data of the two parents (SRA accession is SRP156346).^2^

## Results

### Phenotypic Variation of the Parents and the KN DH Population

**FIGURE 1:**
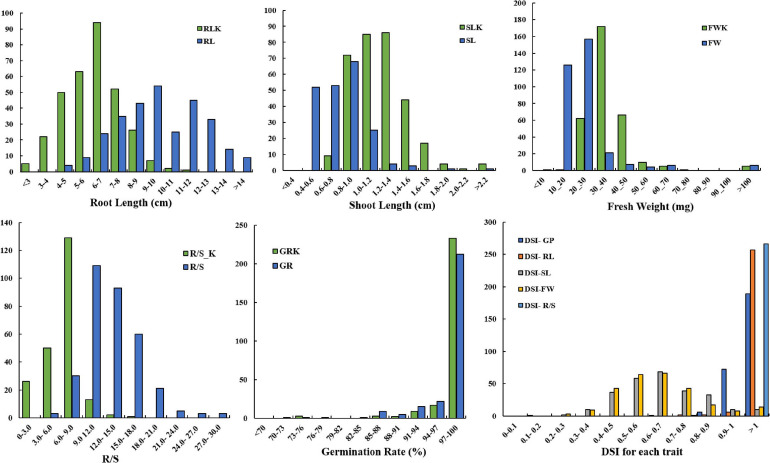
Distribution of germination traits under normal conditions and drought stress conditions. The units on the *x*-axis are the phenotypic values, and the units on the *y*-axis are the number of lines.

The distribution of germination-related traits under drought in the two parents and 300 DH lines in KH populations is shown in Figure 1. Table 1 shows that significant differences were observed between the mean for DSI-RL and DSI-R/S traits of the two parents, while non-significant differences were observed for other traits under drought stress. SL, RL, GP, DSI-RL, and DSI-SL of the parent KenC-8 were higher than those of the parent N53-2, whereas the other traits (FW, R/S, DSI-FW, and DSI-R/S) were low compared to those of N53-2. On the other hand, there was transgressed segregation as well as continuous frequency distribution in all traits under drought stress (Figure 1). Compared to normal conditions, the variation’s average in RL and R/S increased and decreased in SL, while no change in variation’s average was detected in FW between control and drought conditions (Figure 1).

**TABLE 1:** Phenotypic performance of drought tolerance traits in two parents and the DH population.

Traits	Parents		DH populations			
	Ken C-8	N53-2	Mean	Min	Max	SD
GP (%)	100	96	0.89	0.78	1.00	0.40
RL (cm)	11.08	10.37	11.46	4.02	18.91	2.29
SL (cm)	0.89	0.72	1.16	0.43	1.90	0.23
FW (g)	0.02	0.03	0.03	0.01	0.06	0.01
R/S	12.45	14.40	13.54	4.72	28.36	4.15
DSI-GP (%)	1	0.96	0.99	0.01	1	0.07
DSI-RL (%)	2.29	1.58*	1.72	0.72	4.17	0.51
DSI-SL (%)	3.17	1.65	0.66	0.20	3.18	0.24
DSI-FW (%)	0.67	0.77	0.63	0.24	2.76	0.21
DSI-R/S (%)	0.72	0.95*	2.80	0.72	10.73	1.20

GP, germination percentage; RL, root length; SL, shoot length; FW, fresh weight; R/S, root-to-shoot length ratio; DSI, drought stress index. *Differences between two parents for these traits were significant.

### Correlation Among Seedling Traits Under Control and Drought Conditions

As shown in Table 2, a significantly positive correlation was observed for a pair of traits between control and drought stress. R/S was significantly and positively correlated with GP and RL; further analysis revealed that it was significantly and negatively correlated with SL and FW under drought stress. RL was significantly and positively correlated with R/S and slightly positively correlated with GP, while it was significantly and negatively correlated with DSI-RL and DSI-R/S under drought conditions. SL was significantly and negatively correlated with GP, RL, D/S, and DSI-SL, while it was significantly and positively correlated with DSI-R/S under drought stress. DSI-GP was significantly and positively correlated with RL and R/S under drought conditions. DSI-RL was significantly and positively correlated with all measured traits under drought stress. DSI-SL was significantly and positively correlated with RL and SL, while it was negatively correlated with R/S under drought. In addition, DSI-R/S was significantly and positively correlated with RL and R/S, while it was significantly and negatively correlated with R/S. Finally, DSI-FW was significantly and positively correlated with FW, while it was slightly positively correlated with GP and RL under drought stress.

**TABLE 2:** Phenotypic correlation between traits measured under control and drought conditions.

	GP	RL	SL	FW	R/S	DSI-GP	DSI-RL	DSI-SL	DSI-R/S	DSI-FW
GP	**0.034**	0.107	−0.149*	0.110	0.169**	0.321**	0.206**	0.055	0.094	0.012
RL	−0.079	0.370**	−0.182**	0.168	0.433**	0.173**	0.411**	0.280**	0.143*	0.040
SL	−0.036	−0.076	0.159**	0.063	−0.150*	−0.025	0.167**	0.773**	−0.270**	−0.040
FW	0.129	−0.267	−0.106	**0.630**	−0.118	−0.051	0.436**	0.047	0.136	0.996**
R/S	−0.034	0.303**	−0.281**	0.153	0.442**	0.151*	0.220**	−0.249**	0.357**	0.062
DSI-GP	−0.887**	0.077	0.013	0.214	0.055	**NA**	0.079	−0.010	0.086	−0.001
DSI-RL	−0.044	−0.640**	−0.011	−0.100	−0.394**	0.079	**NA**	0.145*	0.657**	0.137*
DSI-SL	−0.020	0.047	−0.431**	0.173	0.334**	−0.010	0.145*	**NA**	−0.475**	−0.018
DSI-R/S	−0.025	−0.485**	0.482**	−0.20	−0.568**	0.086	0.657**	−0.475**	**NA**	0.102
DSI-FW	−0.06	−0.102	−0.030	−0.293	−0.062	−0.001	0.137*	−0.018	0.102	**NA**

Traits measured under control are shown horizontally and those measured under drought stress are shown vertically. The cross-correlation coefficients between drought and control are shown in the diagonal in boldface. *Significant at the 0.05 level. **Significant at the 0.01 level.

### QTL Mapping for Germination-Related Traits Under Control and Drought Conditions

**FIGURE 2:**
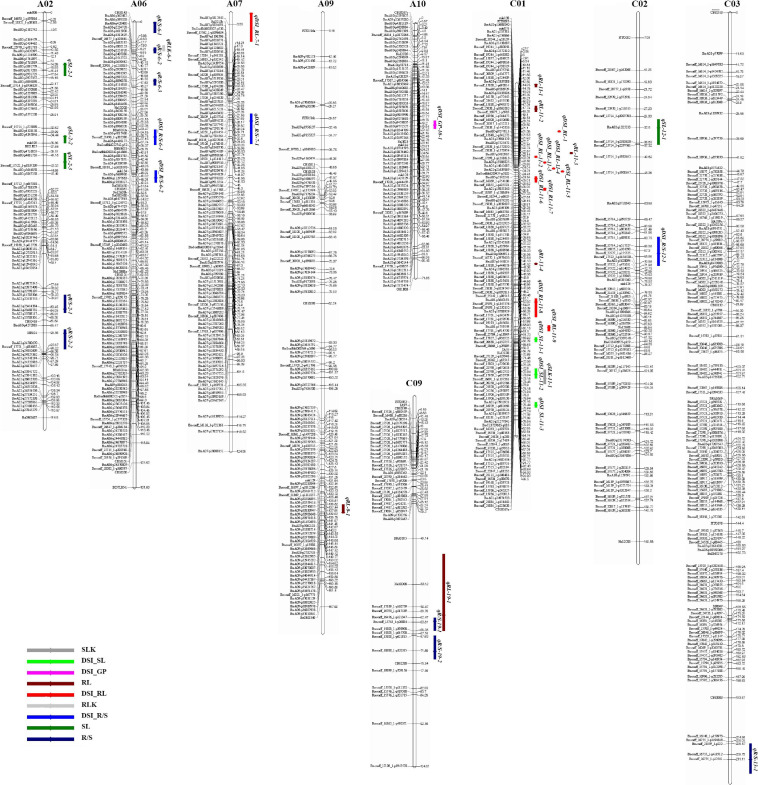
The locations of putative QTLs for drought tolerance. The numbers of linkage groups are shown at the top, and the genetic distance (cM) and markers are indicated on the **left** and **right**, respectively. The QTLs for germination traits are signed on the right of linkage groups.

Using the high-density SNP linkage map and phenotypic data of seedling traits under control and drought conditions, a total of 39 QTLs were detected in KN DH populations (Supplementary Table 1). Thirty-nine identified QTLs were integrated into 36 consensus QTLs, of which 2 QTLs were detected under control, 16 QTLs were detected for individual traits under drought stress, and 18 QTLs controlled DSI for the five individual traits (Figure 2 and Table 3). 14 QTLs were located on the A sub-genome, while the remaining 22 QTLs were located on the C sub-genome. According to the integration results, three unique QTLs (uq-A6, uq-C1, and uq-C9) were detected on A06, C01, and C09, respectively (Table 4).

**TABLE 3:** Detailed information of consensus QTLs.

Trait^a^	QTL	LGs^b^	Peak (cM)	Range (cM)	Flanking markers	LOD	*R*^2c^	Add^d^
RL	qRL-9-1	A09	141.31	139–141.5	Bn-A09-p31730182_Bn-scaff_16197_1-p13988	2.80	4.08	–0.47
	qRL-11-1	C01	7.01	6.2–7.9	Bn-A10-p13608040-niab071	2.63	4.08	0.63
	qRL-11-2	C01	19.51	19.3–19.8	Bn-A09-P20133806_Bn-scaff_15838_1-p868199	3.23	5.50	–0.83
	qRL-11-3	C01	50.81	50.6–51.1	Bn-A01-p3640191_Bn-scaff_18712_1-p329749	4.56	7.45	0.78
	qRL-19-1	C09	61.81	44.5–66.3	Bn-scaff_17174_1-p489628_Bn-A10-p8487448	3.45	5.04	0.51
SL	qSL-2-1	A02	14.71	14.2–14.9	Bn-A06-p5762475_Bn-A02-p2931725	5.64	8.33	–0.08
	qSL-2-2	A02	35.1	34.7–36.9	Bn-A02-p4345046_B012M04	4.42	6.55	0.08
	cqSL-2-3	A02	43.55	41.61–45.48	Bn-A02-p4716855_Bn-A02-p5933593	4.62	6.90	0.07
	qSL-12-1	C02	32.51	30.10–37.30	Bn-scaff-15714_1-p3007703_Bn-scaff-15714_1-p1949380	2.62	3.57	–0.08
RLk	qRLk-6-1	A06	7.91	6.60–8.20	Bn-A02-p362402_Bn-A02-p1612900	3.48	5.56	–1.42
SLK	qSLk-11-1	C01	83.21	80.8–84.8	Bn-scaff_17233_1-p19952_Bn-scaff_17515_1-p27159	3.85	5.86	–0.11
R/S	cqR/s-2-1	A02	86.89	85.0–88.77	Bn-A02-p23708678_Bn-A02-p23856500	3.79	5.07	–0.98
	qR/s-6-1	A06	0.01	0.00–3.10	CB10143_BRMS-027a	2.92	3.93	–0.87
	qR/s-6-2	A06	7.91	7.20–8.80	Bn-A06-p506366_Bn-A06-p1652088	4.71	6.24	–1.09
	qR/s-6-3	A06	16.61	16.10–17.90	Bn-scaff_20901_1-p1210835_Bn-A06-p2938890	3.63	4.85	–0.95
	qR/s-13-1	C03	211.11	206.60–215.10	Bn-scaff_23098_1-p304246_FIT0347	2.78	3.62	0.82
	qR/s-19-1	C09	63.51	62.50–66.20	Bn-scaff_16456_1-p121347_Bn-scaff_15808_1-p398909	5.24	6.98	1.14
	qR/s-19-2	C09	72.01	67.60–74.30	Bn-scaff_16269_1-p529695_CB10288	4.03	5.41	0.99
DSI_GP	qDSI_GP-10-1	A01	28.61	27.40–29.60	Bn-A10_p11375260_Bn-A10_p11420261	3.49	5.60	0.28
DSI_RL	qDSI_RL-7-1	A07	0.01	0.00–8.20	Bn-A07_p1415413_MR166	3.74	5.30	1.41
	qDSI_RL-11-1	C01	17.31	17.10–17.40	Bn-scaff_15838_1-p724409_Bn-scaff_15838_1-p740881	3.27	5.17	1.27
	qDSI_RL-11-2	C01	20.61	20.30–20.70	Bn-scaff_23432_1-p155530_Bn-scaff_15835_1-p949372	3.99	5.97	1.36
	qDSI_RL-11-3	C01	22.21	22.00–22.40	Bn-scaff_17109_1-p945905_Bn-A08_p3184233	4.59	6.55	1.48
	qDSI_RL-11-4	C01	23.91	23.80–24.10	Bn-A07_p7035715_Bn-scaff_15838_1-p1554155	3.96	5.86	1.37
	qDSI_RL-11-5	C01	24.71	24.60–25.00	Bn-A05_p14042687_Bn-A02_p21639869	3.90	5.62	1.40
	qDSI_RL-11-6	C01	26.81	26.10–27.80	Bn-A05_p2143241_Bn-A01_p19855343	3.92	5.65	1.35
	qDSI_RL-11-7	C01	29.61	29.50–30.10	Bn-scaff_15838_1-p2039607_Bn-A02_p849709	4.15	5.95	1.37
	qDSI_RL-11-8	C01	61.61	60.60–65.90	Bn-scaff_17827_1-p1020750_SA54	3.65	5.15	1.17
	qDSI_RL-11-9	C01	68.71	67.10–69.30	Sa49_Bn-scaff_16162_1-p65545	3.60	5.09	1.18
DSI_SL	qDSI_SL-11-1	C01	72.21	71.60–73.00	Bn-scaff_22790_1-p810752_Bn-scaff_24613_1-p1327	3.16	4.76	0.56
	qDSI_SL-11-2	C01	82.11	80.50–83.20	Bn-scaff_17233_1-p19952_Bn-scaff_17515_1-p576696	4.86	7.23	0.67
	qDSI_SL-11-3	C01	90.81	90.00–91.50	Bn-scaff_22795_1-p12914_Bn-scaff_15936_1-p212533	4.70	6.99	0.66
DSI_R/S	qDSI_R/S-6-1	A06	33.61	30.70–34.20	Bn-A06_p3708256_Bn-A06_p3986320	3.90	5.60	–2.88
	qDSI_R/S-6-2	A06	41.91	41.00–42.70	Bn-A06_p4597746_Bn-A06_p4337637	3.54	5.10	–2.74
	qDSI_R/S-7-1	A07	30.61	28.50–37.10	Bn-A07_p6217329_Bn-A07_p8854569	3.77	3.95	2.43
	qDSI_R/S-12-1	C02	68.10	63.40–71.50	Bn-scaff_15714_1-p633949_Bn-scaff_17522_1-p1483337	3.01	4.29	2.67

*^*a*^Trait: three salt tolerance indicators. ^*b*^LGs: chromosome on which QTL was located. ^*c*^*R*^2^: variation accounted for by each putative QTL. ^*d*^Add: additive effect.*

**TABLE 4:** Unique QTLs controlling drought tolerance-related traits.

Unique QTL	Peak (cM)	Range (cM)	Consensus QTL	LGs^a^	Confidence intervals	LOD	*R*^2b^	Add^c^
uq-A6	7.91	7.3–8.5	qR/s-6-2	A06	Bn-A06-p506366-Bn-A06-p1652088	4.71	6.24	–1.09
			qRLk-6-1		Bn-A02-p362402-Bn-A02-p1612900	3.48	5.56	–1.42
uq-C1	82.66	80.5–84.8	qDSI_SL-11-2	C01	Bn-scaff_17233_1-p19952-Bn-scaff_17515_1-p576696	4.86	7.23	0.67
			qSLk-11-1		Bn-scaff_17233_1-p19952-Bn-scaff_17515_1-p27159	3.85	5.86	–0.11
uq-C9	63.46	61.6–65.3	qR/s-19-1	C09	Bn-scaff_16456_1-p121347-Bn-scaff_15808_1-p398909	5.24	6.98	1.14
			qRL-19-1		Bn-scaff_17174_1-p489628–Bn-A10-p8487448	3.45	5.00	0.51

*^*a*^LGs.: chromosome on which QTL was located. ^*b*^*R*^2^: variation accounted for by each putative QTL. ^*c*^Add: additive effect.*

For QTL analysis under control conditions, two QTLs (qRLK-6-1 and qSLK-11-1) were detected for RL and SL under control conditions on A06 and C01, respectively. The two QTLs have a negative effect on RL and SL, accounting for 5.56 and 5.86% of phenotypic variation (PV), respectively. No QTLs were detected for R/S, FW, and GP under control conditions.

For QTL analysis under drought conditions, a total of 16 QTLs were detected for three different traits (RL, SL, and R/S) under drought conditions (Supplementary Table 1). Compared to other chromosomes, A02 and C01 contained 19 QTLs associated with more than one trait. Seven QTLs associated with R/S trait under drought stress were located on A02, A06, C03, and C09. cqR/S-2-1 was detected on A02 and accounted for 6.39% of PV. Three QTLs (qR/S-6-1, qR/S-6-2, and qR/S-6-3) were detected on A06 and accounted for 3.13, 6.24, and 4.85% of PV, respectively. Three other QTLs for R/S, qR/S-13-1, qR/S-19-1, and qR/S-19-2 that accounted for 3.62, 6.98, and 5.41% of total PV were located on C03 and C09, which have a positive effect on the R/S ratio. For RL, five QTLs were mapped to three chromosomes. Three QTLs (qRL-11-1, qRL-11-2, and qRL-11-3) were located on C01 and showed 2.63, 5.50, and 7.45% of PV, respectively. In addition, two QTLs associated with RL (qRL-9-1 and qRL-19-1) were detected on A09 and C09, accounting for 2.80 and 3.45% of the PV, respectively. Four QTLs were detected for SL, of which three QTLs (qSL-2-1, qSL-2-2, and qSL-2-3) were located on A02, accounting for 5.64, 4.42, and 4.60% of PV, respectively. No QTLs were detected for FW and GP under drought conditions.

For QTL mapping for DSI, 18 QTLs affecting DSI of four traits (GP, RL, SL, and R/S) were identified on different chromosomes, including A06, A07, A10, C01, and C02 (Supplementary Table 2), and no QTL was detected for DSI-FW. The distribution of these QTLs was as follows: one QTL for GP was detected on A10 and accounted for 5.6% of PV. Ten QTLs for RL (one QTL on A07 and nine QTLs on C01) and PV ranged from 5.09 to 6.55%. Three QTLs for SL on C01 with PV ranged from 4.76 to 7.23%. In addition, four QTLs for R/S on A06, A07, and C02 with PV ranged from 3.95 to 5.60%.

### Candidate Genes in Response to Drought Stress

According to the co-linearity between genetic and physical maps of *B. napus*, 256 candidate gene homologs with 234 genes involved in drought tolerance in *Arabidopsis thaliana* were detected (Supplementary Table 3). According to the data from re-sequencing of the two parents, 128 candidate genes have SNP/InDel variations between the two parents (Supplementary Table 4). The candidate genes were located within 36 QTLs on both the A and C genome and associated with three individual traits (RL, SL, and R/S) and DSI for all individual traits except FW.

Based on gene function annotation and genomic variation analysis, many important candidate genes involved in drought-related stress were identified. *BnaC01g35030D* that was homologous to the *A. thaliana* gene *AT1G18080* was located on chromosome C01 within the two QTLs (qDSI_RL-11-1 and qDSI_SL-11-3) associated with both RL and SL under drought stress, which encoded a protein involved in cellular responses to abscisic acid. *BnaC01g32600D* was located on chromosome C01 within the two DSI-QTLs (qDSI_RL-11-2 and qDSI_SL-11-3) associated with both RL and SL under drought stress and encoded for an ortholog of the *A. thaliana* gene *AT3G20340* involved in oxidative stress. *BnaA06g02020D* was located within QTLs qR/S-6-2 and qRLK-6-2, associated with R/S under drought and RL under control conditions, respectively, and orthologous to the *A. thaliana* gene “LysM-containing receptor-like kinase 3 (*LYK3*),” which functions in the abscisic acid-activated signaling pathway. *BnaC01g34530D* in QTL qSLK-11-1 was associated with SL under control, and it was orthologous to the *A. thaliana* gene “HALOTOLERANCE DETERMINANT 3 (*HAL3A*).” *BnaC03g32780D* within QTL qR/S-13-1 on chromosome C03 associated with R/S under drought was orthologous to the *A. thaliana* gene “ABI BINDING PROTEIN4 (*AFP4*).” *BnaC03g37030D* in QTL qR/S-13-1 was orthologous to the *A. thaliana* gene “DEHYDRATION-RESPONSIVE ELEMENT BINDING PROTEIN 2 (*DREB2*).” *BnaC03g44440D* was located in QTL qR/S-13-1 associated with R/S under drought conditions and orthologous to the *A. thaliana* gene “RING/U-box superfamily protein (*XERICO*).” *BnaC03g45915D* within QTL qR/S-13-1 was orthologous to the *A. thaliana* gene “LOW-TEMPERATURE-INDUCED 65, (*RD29B*).” *BnaC03g45990D* in QTL qR/S-13-1 associated with R/S under drought stress was an ortholog of the *A. thaliana* gene “COLD REGULATED 413 PLASMA MEMBRANE1 (*COR413-PM1*).” *BnaC03g46570D* was detected in QTL qR/S-13-1, orthologous to the *A. thaliana* gene “ASCORBATE PEROXIDSE 3 (*APX3*).” *BnaC03g49530D* in QTL qR/S-13-1 associated with R/S under drought conditions and orthologous to the *A. thaliana* gene “ABA REPRESSORE1, (*ABR1*).” *BnaC03g64160D* was detected within QTL qR/S-13-1 and was an ortholog of the *A. thaliana* gene “A. THALIANA MYB 4 (*ATM4*).” *BnaC09g24550D* in QTLs qR/S-19-1 and qRL-19-1 was associated with R/S and RL under drought stress, respectively, and orthologous to the *A. thaliana* gene “A. THALIANA INDUCAR OF CBP EXPRESSION 1 (*ATICE1*).” Two genes, *BnaC09g27300D* and *BnaC09g27320D*, were also located within QTL qR/S-19-1 and RL-19-1, and orthologous to the *A. thaliana* genes “RUBISCO ASSEMPLY FACTOR 2 (*RAF2*)” and “*ATYY1*”, respectively. *BnaC09g36060D* was detected in QTL qR/S-19-2 associated with R/S and orthologous to the *A. thaliana* gene “METHYLTHIOALKYLMALATE SYNTHASE 2 (*MAM2*)” (Supplementary Table 3). These important candidate genes provided insights into drought tolerance in *B. napus*, and their function could be verified further though a trans-gene method in the future.

## Discussion

### PV Under Drought Stress

Drought is one of the crises we are facing in the present time; therefore, it is necessary to develop new drought-tolerant crops with high seed germination vigor. Drought tolerance of crops is a very complex phenomenon because it is controlled by interaction between several factors, and some factors are only activated under drought stress. So, it is very difficult to evaluate these factors under non-stressed conditions. One of the effective methods to evaluate drought tolerance in crops is through the using of morphological traits under drought stress. In the present study, we used a number of morphological indexes to determine drought tolerance of *B. napus* and QTLs related to drought tolerance. It was revealed that the R/S was sensitive to drought stress, and it was consistent with the results of Dhanda et al. (2004) in wheat. On the other hand, higher correlation was also detected between R/S and DSI and other morphological traits (Table 2). In this study, data in Table 1 and Figure 1 illustrated that the parent N53-2 showed higher FW, R/S, and DSI than that of the parent KenC-8 under drought stress, which suggested that N53-2 was more tolerant to drought. We assumed that the use of DSI could help us in breeding and population screening under drought stress conditions. Moreover, all phenotypic traits showed transgressive segregation in the DH population, which suggested that the two parents contain all +Ve or -Ve alleles, which coincided with the results obtained in *B. napus* by Li et al. (2014). Here, we want to point out that R/S might be considered as an important phenotypic trait due to its sensitivity to drought stress; this finding in consistent with the results in wheat (Dhanda et al., 2004; Li et al., 2014). R/S was positively correlated with GP, RL, and DSI-SL under drought stress, while in the case of control, it showed a negative correlation with GP and SL; the results suggested that *B. napus* is likely to resist the effect of drought through increasing water uptake and reducing its loss. The increase in RL was essential to enhance water uptake, and the decrease in SL resulted in water loss (Dhanda et al., 2004; Ruta et al., 2010). In addition, Poorter and Remkes (1990) reported that, under stress conditions, the shoot biomass of plants decreased compared to roots, which relatively increased. As mentioned above, the R/S can be used as an important basis for assessing the ability of plants to withstand drought stress in the early growth stages.

### The Genetic Bases and QTLs for Drought Tolerance

In the last decades, one of the most important approaches to clarifying quantitative trait crisis in plants was the use of molecular marker techniques for QTL analysis. Plant populations with different genetic structures have been developed to achieve these objectives. QTL analysis was performed as a response to biotic and abiotic stresses in many economically important crops, such as in peanut and wheat (Zhao et al., 2016; Wang et al., 2018). In the present study, the DH population named KN population was phenotyped and the QTL analysis was performed under both control and drought conditions, and some QTL/genes that controlled drought tolerance in *B. napus* were identified. In response to drought stress, many QTLs associated with drought tolerance were identified in tomato and wheat (Foolad et al., 2003; Wang et al., 2009) during seed germination and the early seedling stage, which indicated that a large number of genes are involved in drought tolerance mechanisms (Qie et al., 2014). In this study, a large number of QTLs related to drought resistance distributed on nine chromosomes in *B. napus* were detected. The locations of QTLs for some drought tolerance traits indicated that these traits might have smaller genetic bases. For example, many QTLs related to drought tolerance that contributed to different traits were detected in the same regions, such as qDSI_RL-11-1 and qDSI_SL-11-3, qDSI_RL-11-2 and qDSI_SL-11-3, and qDSI_RL-11-4 and qDSI_SL-11-3 on C01. In addition, qR/S-19-2 and qRL-19-1 were on C09, which suggests the close relationships between these traits and the probability of the presence of a single gene controlling more than one trait in this region; these results are consistent with results obtained under salinity stress in *B. napus* by Lang et al. (2017). Therefore, according to the present results, we considered that there are several key loci that control drought tolerance in *B. napus* (Table 3).

Several QTLs related to important drought-tolerant traits were detected on C01; therefore, this chromosome deserves specific importance in drought resistance in *B. napus*. For example, C01 carries QTL qDSI_SL-11-3 for SL and qDSI_RL-11-1 for RL, both of them adjoined with *BnaC01g35030D* (ortholog with *A. thaliana AT1G18080*) and were involved in abscisic acid stimulus. Two QTLs, qDSI_RL-11-2 and qDSI_SL-11-3, were located in the same position on C01 and associated with the *BnaC01g32600D* gene, which was orthologous to the gene for oxidative stress. Moreover, qDSI_RL-11-4 and qDSI_SL-11-3 were also located near each other and associated with *B. napus* gene *BnaC01g30750D* that is orthologous to *ICE1*, which is involved in ABA signaling and cold stress. However, QTLs qRL-11-3 for RL and qDSI_RL-11-2 affecting DSI were detected in the same region on C01. These results suggested the importance of these regions on chromosome C01 for drought tolerance.

### Candidate Genes in Response to Drought Stress

The problem of selecting effective genes from hundreds of genes within QTLs is the biggest challenge, especially with the presence of many unfunctional genes (Gudys et al., 2018). For this reason, many techniques were used to help in the selection of target genes within QTLs, such as the ortholog of these genes to accessible model species and the upregulation/downregulation of the candidate genes when expressed in proteomic or transcriptional levels and based on the representation of the genes in specific physiological or biochemical pathways (Monclus et al., 2012; Bargsten et al., 2014; Kumar et al., 2017; Gudys et al., 2018). In this study, 256 genes were identified as candidate genes within 36 consensus QTLs. The transgressive segregation has been shown in the DH population due to the variation between the two parents detected in 128 genes. Based on the function annotation of the candidate genes, many genes orthologous to *A*. *thaliana* genes were associated with drought tolerance. *BnaC03g44440D* was located in QTL qR/S-13-1 that is associated with R/S under drought conditions and orthologous to the *A. thaliana* gene *XERICO*. Ko et al. (2006) reported that upregulation of *XERICO* improved drought tolerance in *A. thaliana*. *BnaC03g37030D* was located in QTL qR/S-13-1 and orthologous to the *A. thaliana* gene *DREB2*. Previous studies have reported that *DREB* overexpression enhanced drought tolerance in many plants such as *A. thaliana*, tobacco, and apple (Chen et al., 2016; Liao et al., 2017; Sharma et al., 2019). In this study, the gene *BnaA02g09290D* within QTL qSL-2-4 was orthologous to the *A. thaliana* gene *MAPKKK15*, which was reported to be involved in the drought tolerance mechanism in potato (Pieczynski et al., 2018). The two genes *BnaC09g27300D* and *BnaC09g27320D* were located within QTL qR/S-19-2 and qRL-19-1 and orthologous to the *A. thaliana* genes *RAF2* and *ATYY1*, respectively. The *AtYY1* gene was expressed under ABA, salt, and dehydration conditions, and overexpression of *AtYY1* decreased ABA sensitivity in *A. thaliana* (Li et al., 2016). Furthermore, in the study conducted by Zhang H. et al. (2015), they reported that the *RAF2* and *SDIR1* complex had an essential role under stress by mediating the ABA signaling pathway. In addition, *BnaC03g45915D* within QTL qR/S-13-1 was orthologous to the *A. thaliana* gene *RD29B*, which was highly expressed under desiccation and played a vital role in drought tolerance (Prerostova et al., 2018). Our results illustrated that a lot of the genes related to drought tolerance in this study were located within QTLs associated with R/S, which supports the importance of this trait in QTL mapping. Also, several genes associated with drought tolerance were detected within the QTL for DSI on chromosome C01, such as *BnaC01g35030D*, *BnaC01g32600D*, and *BnaC01g30750D*, which indicated the role of these genes in drought tolerance in *B. napus*. In the end, we assumed that the above genes that were orthologous to drought stress tolerance genes in *A. thaliana* may be strongly associated with drought tolerance in *B. napus*. Further investigation and validation of these genes are underway to confirm their function in the drought tolerance in *B. napus*.

## Conclusion

To illustrate the complexity of drought stress mechanisms and identification of genes underlying these mechanisms, we have used many genetic and genomic technologies. In this study, we used R/S as a phenotyping trait in QTL mapping and gene identification for drought tolerance at seedling stages in *B. napus*. Few studies used R/S as a phenotyping trait to QTLs in *B. napus* before. According to the QTL analysis, 39 QTLs associated with drought tolerance were identified and integrated to 36 consensus QTLs. Moreover, 256 drought tolerant-related candidate genes were identified, of which 128 genes have SNP/InDel variations between the two parents. In addition, our results indicated that specific regions on chromosome C01 were important for improved drought tolerance in *B. napus* due to the presence of many QTLs associated with drought tolerance. However, further studies are required to further confirm the candidate genes related to drought tolerance in *B. napus.*

## Data Availability Statement

The original contributions presented in the study are included in the article/Supplementary Material, further inquiries can be directed to the corresponding authors.

## Ethics Statement

The authors declare that the experiments comply with the current laws of the country in which they were performed.

## Author Contributions

MG and HC conducted most of the experiments and data analysis for the overall study, and wrote and modified the manuscript. HL and WZ conducted parts of data analysis and provided a guide to the use of the related software. GL and ML designed and conceived the overall study and revised the manuscript. All authors read and approved the final manuscript.

## Conflict of Interest

The authors declare that the research was conducted in the absence of any commercial or financial relationships that could be construed as a potential conflict of interest.

## Footnotes

1 http://www.unep-wcmc.org/medialibrary/2011/10/19/3faf1880/Global-Drylands-FINAL-LR.pdf

2 https://www.ncbi.nlm.nih.gov/

## Supplementary Material

Supplementary Table 1 Detailed information of identified QTLs for germination-related traits under control and drought stress.

Supplementary Table 2 QTL for DSI for different agronomic traits identified under drought stress.

Supplementary Table 3 The candidate genes homologues with 234 genes involved in drought tolerance in *A. thaliana*.

Supplementary Table 4 128 candidate genes have SNP/InDel variations between the two parents.

## Abbreviations

QTL: quantitative trait locus
SNPs: single-nucleotide polymorphisms
InDel: insertion–deletion
DH: double haploid
GP: germination percentage
RL: root length
SL: shoot length
FW: fresh weight
R/S: root-to-shoot length ratio
DSI: drought stress index.
